# Screening mammography beliefs and recommendations: a web-based survey of primary care physicians

**DOI:** 10.1186/1472-6963-12-32

**Published:** 2012-02-06

**Authors:** Shagufta Yasmeen, Patrick S Romano, Daniel J Tancredi, Naomi H Saito, Julie Rainwater, Richard L Kravitz

**Affiliations:** 1 Department of Internal Medicine and Centre for Healthcare Policy and Research University of California Davis School of Medicine, Sacramento, California, USA; 2 Department of Obstetrics Gynaecology University of California Davis School of Medicine, Sacramento, California, USA; 3 Department of Paediatrics and Centre for Healthcare Policy and Research University of California Davis School of Medicine, Sacramento, California, USA

## Abstract

**Background:**

The appropriateness and cost-effectiveness of screening mammography (SM) for women younger than 50 and older than 74 years is debated in the clinical research community, among health care providers, and by the American public. This study explored primary care physicians' (PCPs) perceptions of the influence of clinical practice guidelines for SM; the recommendations for SM in response to hypothetical case scenarios; and the factors associated with perceived SM effectiveness and recommendations in the US from June to December 2009 before the United States Preventive Services Task Force (USPSTF) recently revised guidelines.

**Methods:**

A nationally representative sample of 11,922 PCPs was surveyed using a web-based questionnaire. The response rate was 5.7% (684); (41%) 271 family physicians (FP), (36%) 232 general internal medicine physicians (IM), (23%) 150 obstetrician-gynaecologists (OBG), and (0.2%) 31 others. Cross-sectional analysis examined PCPs perceived effectiveness of SM, and recommendation for SM in response to hypothetical case scenarios. PCPs responses were measured using 4-5 point adjectival scales. Differences in perceived effectiveness and recommendations for SM were examined after adjusting for PCPs specialty, race/ethnicity, and the US region.

**Results:**

Compared to IM and FP, OBG considered SM more effective in reducing breast cancer mortality among women aged 40-49 years (*p *= 0.003). Physicians consistently recommended mammography to women aged 50-69 years with no differences by specialty (*p *= 0.11). However, 94% of OBG "always recommended" SM to younger and 86% of older women compared to 81% and 67% for IM and 84% and 59% for FP respectively (*p = *< .001). In ordinal regression analysis, OBG specialty was a significant predictor for perceived higher SM effectiveness and recommendations for younger and older women. In evaluating hypothetical scenarios, overall PCPs would recommend SM for the 80 year woman with CHF with a significant variation by specialty (38% of OBG, 18% of FP, 17% of IM; *p *= < .001).

**Conclusions:**

A majority of physicians, especially OBG, favour aggressive breast cancer screening for women from 40 through 79 years of age, including women with short life expectancy. Policy interventions should focus on educating providers to provide tailored recommendations for mammography based on individualized cancer risk, health status, and preferences.

## Background

Screening mammography every one to two years for women aged 50 to 69 years has resulted in earlier breast cancer detection, and reduced breast cancer mortality [1,2]. The value of screening mammography for this age group is widely accepted. However, the appropriateness and cost-effectiveness of screening mammography in women younger than 50 and older than 74 years of age is not fully established, leading professional societies to issue conflicting recommendations [3].

The United States Preventive Services Task Force (USPSTF) recommends against routine screening mammography for average-risk younger (ages 40 to 49) women, in favour of "individualized, informed decision making about when to begin screening mammography" and suggests that biennial mammography be encouraged from ages 50-74 [4]. The American Cancer Society (ACS) differs slightly, providing no specific age for stopping and stating that comorbidity is the only qualifying factor for exclusion from screening. The American College of Obstetricians and Gynaecologists (ACOG) [5] recommends mammography every one to two years in women from 40 to 50 years of age and annually after age 50 with no specific age for stopping. The American Geriatrics Society recommends mammography for older women unless they are unlikely to survive 5 years or have significant comorbidities that would preclude breast cancer treatment [6,7].

Primary care physicians (PCPs) play a critical role in recommending age-appropriate cancer screening to eligible patients, and their acceptance of practice guidelines is essential in translating recommendations into improved health outcomes. However, when practice guidelines are unclear, physicians and patients can become confused [8,9]. Lack of consensus on screening mammography guidelines for younger and older women allows physicians to come to different conclusions regarding when to use mammography [10]. Such uncertainties can lead to underuse of cancer screening in healthy older women with substantial life expectancies, and overuse of cancer screening in women with severe comorbidities and limited life expectancies.

Previous studies have identified physician characteristics, including specialty, age, and gender that are associated with the use of screening mammography [11,12]. For example, OBG are more likely to recommend mammography to women 40-49 years of age than general internists and family physicians [13]. However, it is not well understood whether this variation in physician practices is related to variation in clinical practice guidelines, and the influence of those guidelines on primary care physicians of three primary care specialty. The objectives of this study were to explore (1) US primary care physicians' beliefs about the effectiveness of screening mammography before the revised USPSTF guidelines in 2009; (2) their decisions regarding screening mammography in hypothetical clinical case scenarios; and (3) predictors of effectiveness and recommendations for screening mammography in different age categories. We hypothesized that physicians' recommendations for screening mammography for younger and older women are related to their demographic and practice characteristics (particularly specialty, as previously documented), but also to their beliefs about the effectiveness of mammography, and influence of published practice guidelines.

## Methods

### Study design

We surveyed a nationally representative sample of 11,922 PCPs (general internists, family physicians, and obstetricians and gynecologists) in the US between June, 2009 and December, 2009. The sample of physicians was obtained from the American Medical Association's [14] Physician Masterfile [11,13]. A random sample of primary care physicians were invited to participate in a web based questionnaire on screening mammography practices from June 2009 to December 2009. Eligible respondents included physicians younger than 76 years of age, who held an active license to practice medicine, and listed patient care as their primary professional activity. We excluded physicians over 75 years of age, and physicians who were not engaged in patient care.

### Survey instrument

The questionnaire was composed of three parts: (a) 6 single item questions about knowledge and beliefs regarding screening mammography and recommendations in community practice, (b) 8 questions about decision-making for mammography in specific clinical situations, and (c) 16 single item questions on personal background and practice characteristics. The questions in part a of the questionnaire used four-point adjectival scales that included the response categories "not effective", "somewhat effective", "very effective", and "not sure." The perceived influence of USPSTF guidelines on physicians practice was measured using the response categories "not influenced", "somewhat influenced", "extremely influenced", and "not familiar with this guideline." Additional questions described asymptomatic, average risk women in 3 different age categories [40-49 y, 50-69 y, 70-89 y], and asked respondents about their usual advice on starting age and stopping age, for which professional guidelines are unclear or conflicting. "Conflicting" guidelines were defined as different screening mammography recommendations from at least two different organizations. Response items included "never recommend," "rarely recommend", "sometimes recommend", "often recommend," and "always recommend," as appropriate. Written case simulations (vignettes) in part b were designed on the basis of a fractional factorial design that ensures the absence of collinearity. For the clinical vignettes (case scenarios), the dependent variable was the physician's decision to recommend mammography or not, and the independent variables were age (young and old), health status (comorbidities present/absent), and severity and comorbidity burden (associated within life expectancies of < 5 and > = 5 years). Screening mammography decision-making was assessed by asking physicians whether they would recommend or not recommend screening mammography for each hypothetical case; physicians responded "definitely would not recommend," "probably would not recommend," "probably would recommend," or "definitely would recommend."

The questionnaire items and the format were pilot tested for clarity and face validity in two different settings, including primary care physician participants in a health services research seminar at UC Davis and a web based questionnaire e-mailed to primary care colleagues in academic settings.

### Sampling methodology

Using the American Medical Association's (AMA) Physician Masterfile as the sampling frame, we aimed to survey a nationally representative sample of primary care physicians [15] from June 2009 though December 2009. The AMA Masterfile contains demographic and practice-related data on virtually all allopathic and osteopathic physicians in the United States. Obstetricians/gynaecologists (OBG) were included in the sample because they provide preventive services for many women in the United States [16,17]. We asked the AMA to provide overall counts of primary care physicians with accessible email addresses and counts of PCPs with no email addresses, for 3 primary care specialties in the 4 US regions. A total of 261721 PCPs were identified in the database and email addresses were available for (44.7%) 119747. Of those (40.4%) 48378 were family physicians, (43.6%) 52199 general internists, and (16%) 19170 obstetricians/gynaecologists. The sampling frame was stratified by physician specialty (IM, FP and OBG) and years in practice (1-9, 10-20 and > 20). Systematic random sampling was performed after sorting the sampling frame by U.S. Census region (Northeast, Midwest, South, and West) to ensure adequate representation of primary care physicians in each Census region (Northeast, Midwest, South, and West). OBG and the US regions where < = 30% physicians were accessible by email were oversampled at a rate of approximately 2.5 to achieve appropriate representation of physicians by specialty (IM, FP and OBG) in all regions. The probability of selection for physicians in each specialty was proportional to the specialty's representation in the U.S. physician population. Population counts and sample specifications were provided to the AMA. Sample variables requested from the AMA are displayed in (Table 1).

**Table 1:** Responding primary care physicians' demographic and practice characteristics

Primary care specialty	Family Physicians		General Internal Medicine		Obstetrics and gynaecology				
	**FP**		**IM**		**OBG**		**Total**		
	**n = 271 (41.5%)**		**n = 232 (35.5%)**		**n = 150 (23.0%)**		**n = 653**		***P- value***

**Race/Ethnicity**									
White	213 (79)		164 (71)		120 (81)		497 (76)		0.002
AA	8 (3)		12 (5)		4 (3)		24 (4)		
Hispanic	15 (6)		2 (1)		3 (2)		20 (3)		
Asian	30 (11)		51 (22)		21 (14)		102 (16)		
Other	5 (2)		3 (1)		1 (1)		9 (1)		
**Gender**									
Male	161 (59)		136 (59)		71 (47)		368 (56)		0.04
Female	110 (41)		96 (41)		79 (53)		285 (44)		
**Age in years**									
25-44	103 (38)		93 (40)		48 (32)		244 (37)		0.403
45-54	82 (30)		71 (31)		53 (36)		206 (32)		
55-64	71 (26)		58 (25)		36 (24)		165 (25)		
65 +	15 (6)		10 (4)		12 (8)		37 (6)		
**Number of years in practice**									
1~9 yrs	106 (39)		92 (40)		56 (37)		254 (39)		0.57
10~20 yrs	103 (38)		80 (34)		49 (33)		232 (36)		
> = 21 yrs	62 (23)		60 (26)		45 (30)		167 (26)		
**US Region**									
North East	52 (19)		76 (33)		39 (26)		167 (26)		< 0.001
Mid West	67 (25)		55 (24)		19 (13)		141 (22)		
South	63 (23)		58 (25)		38 (25)		159 (24)		
West	89 (33)		43 (19)		54 (36)		186 (28)		
**Percent of female patients seen per week (mean)**									
New patients	21%		25%		37%		26%		< 0.001
**Had a mammogram (Female physicians only)**									n = 238
Yes	77 (71)		68 (72)		58 (73)		203 (72)		
No	32 (29)		27 (28)		21 (27)		80 (28)		
**Personal History of breast cancer (Female physicians only)**									n = 282
Yes	7 (6)		3 (3)		0 (0)		10 (4)		
No	103 (94)		92 (97)		77 (100)		272 (96)		
**Family History of breast cancer**									n = 472
Yes	60 (30)		58 (37)		45 (38)		163 (35)		
No	137 (70)		99 (63)		73 (62)		309 (65)		
Percent of time spent week *									
	Mean	SD	Mean	SD	Mean	SD	Mean	SD	
Patient care	83	23	78	26	79	21	80	24	
Teaching/research	10	16	15	20	12	16	12	17	
Administration	13	15	14	17	13	13	13	15	
Other	2	6	6	16	3	13	4	12	

P is significant for < 0.05.

* Data are presented as mean and standard deviation.

Anticipating a 10% response rate we estimated that a total of 11,922 would be sufficient to provide 80% power to find a 10% difference between physician specialties including family physicians, general internists and obstetricians/gynaecologists. Accordingly primary care physicians were emailed a Broadcast letter from the AMA describing the objectives of the survey, with a link to a secure Internet website. Instructions were provided on how to complete the survey if the respondent chose to open the link to the survey. The survey took approximately 15 minutes to complete. The cover letter stressed the importance of breast cancer screening recommendations and comorbidities to the nation's public health. A financial incentive of $5 for the first round was subsequently increased to $ 20 in follow-up rounds to improve the response rate. The study was approved by the Institutional Review Board of the University of California, Davis.

### Statistical methods

Demographic characteristics, practice characteristics, and responses to questionnaire items were examined by physician specialty. Differences in response distributions between physician specialties were considered statistically significant at the *p *< 0.05 level. We tested the statistical significance of differences between ratings across specialties using analysis of variance.

We tested bivariate associations between the independent variables and outcomes including; physicians perceived screening mammography effectiveness in decreasing mortality from breast cancer, influence of guidelines, and screening mammography recommendations for women in 3 age groups using ANOVA for categorical variables. We then performed ordinal logistic regression to analyze the association of response scores at 1 level increase in the odds ratios for each outcome of interest) with significant physician and practice characteristics. For physician's beliefs regarding perceived effectiveness, the outcome variable was scored 1 for "not effective", 2 for "somewhat effective", and 3 for "very effective": respondents who said, "not sure" were excluded due to small numbers. To analyze the influence of USPSTF guideline on physicians practice, the outcome variable was scored 1 for "not influenced", 2 for "somewhat influenced", and 3 for "extremely influenced"; respondents who said, "not familiar with guidelines" were excluded due to small numbers. To analyze screening mammography recommendations, we compared "always" and "often" to "sometimes", "rarely", and "never" for 40-49 and 70-80 years old women. For 50-69 years old women, we compared "always" to "often", "sometimes", rarely", and "never" because no IM physician answered "often". The rates for missing values were less than 5% for item specific response and were excluded from the analysis.

After accounting for undeliverable emails, we compared physicians who responded later in the survey administration period, after the second and third Med E-Mail Broadcast and higher incentives, to physicians who responded to the first Med E-Mail Broadcast and lower incentives, to explore the potential for response bias. Previous studies suggest that late or marginal respondents to a survey are often similar to non-respondents, in comparison with early respondents [18-20]. Differences in demographic and practice characteristics between early and late respondents, and their association with mammography practices and recommendations, were examined. No information was available about nonrespondents.

## Results

Of the 11, 922 physicians invited to complete a web-based questionnaire, 11103 (93%) were delivered, and 11% were undelivered. Of the 11,103 only (2%) 227 opened the survey at first e-mail broadcast and 1% completed the survey. In three subsequent e-mail broadcasts when the incentive was increased to $ 20, the cumulative response rate increased to 7% with 2% click through and 5% completing the survey. The initial survey was emailed in June 2009, and the survey was closed in December 2009.

A total of 684 physicians completed the survey, of whom 271 (41%) were family physicians (FP), 232 (36%) were general internal medicine physicians (IM), 150 (23%) were obstetrician-gynaecologists (OBG), and 31 did not practice primary care and were eliminated from further analysis. Overall respondents were predominantly white (76%), male (56%) and 79% provided patient care. Physicians' age, number of years in practice, and the percent of female patients seen in a typical week did not vary by primary care specialty (Table 1).

### Description of respondents and perceived effectiveness of screening mammography

**Figure 1:**
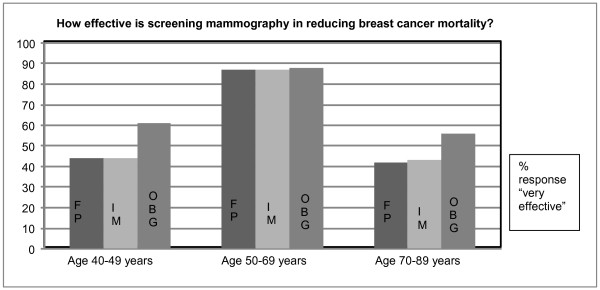
**Primary care physicians' perceived effectiveness of screening mammography for average-risk women by age categories**. How effective is screening mammography in reducing breast cancer mortality?

The proportion of family physicians and internal medicine who believed that screening mammography is very effective in reducing breast cancer mortality was 44% for women aged 40-49 years, 87% for women 50-69 years, and 42% for women 70-79 years of age (Figure 1). However, obstetricians and gynaecologists indicated stronger belief in the effectiveness of screening mammography in reducing breast cancer mortality by 61% in younger (40-49 year old) and 56% older (70 years or over) women.

Physician's age, gender, years in practice, personal or family history of breast cancer were not associated with perceived mammography effectiveness for women across all age categories.

### Influence of published USPSTF guidelines and screening mammography recommendations by patient's age and health status

Overall 45% of respondents rated the U.S. Preventive Services Task Force (USPSTF) guidelines as "extremely influential," but endorsement of this statement was higher among FP (53%) and IM (47%) than among OBG (25%) (*P = *< .001 for both comparisons). In comparison with FP and IM, OBG were more likely to describe American Cancer Society (ACS) (*p *= 0.07) and American College of Obstetricians and Gynaecologists guidelines (ACOG) (*p *= < 0.001) to be "very influential" on their practice.

**Figure 2:**
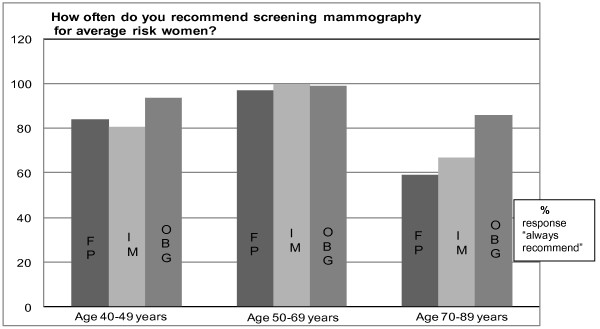
**Primary care physicians' recommendations for screening mammography**. How often do you recommend screening mammography for average-risk women?

Greater than 95% of respondents indicated that they (often and always responses were combined: "always") "always" recommend screening mammography to average risk women aged 50-69 years, with no differences by physician specialty *(p = 0.11)*. However, OBG differed from IM and FP in their recommendations for younger women: 94% of OBG "always" recommended mammography to women 40-49 years versus 81% of IM and 84% of FP (*p = *< .001 for both comparisons). Similarly, a significantly higher proportion of OBG (86%) indicated "always" recommending screening mammography to older women (aged 70-89 years), compared to 67% for IM and 59% for FP (*p = *< .001 for both comparisons). None of these differences between IM and FP was statistically significant (Figure 2).

Physicians' clinical decision-making regarding recommending a screening mammogram (yes/no) for hypothetical case vignettes showed that overall 37% of physicians would recommend mammography for a 50 year old woman with unresectable non-small cell lung cancer, and 21% for an 80 year old woman with congestive heart failure from ischemic cardiomyopathy who has dyspnoea with ordinary activities. However, OBG (37%) differed significantly from IM (17%) and FP (16%) in recommending mammography in hypothetical case-situations with limited life expectancies of < 5 years (*p = < 0.001*). There were no significant differences across physician specialties regarding mammography recommendations for asymptomatic 55 and 70 year old women (Table 2).

**Table 2:** Primary care physicians' responses to clinical vignettes by primary care specialty

Primary care specialty	Family Physicians	General Internal Medicine	Obstetrics and gynaecology				
	**FP**	**IM**	OBG*	**Over all**	**IM vs OBG**	**FP vs OBG**	**FP vs IM**

**Would order screening mammogram for following case scenarios**.......	n (%)	n (%)	n (%)	*P- value*			
**Healthy 70 year old woman**	245 (90)	223 (96)	143 (95)	0.024	0.795	0.088	0.013
**50-year-old with unresectable non-small cell lung cancer**	102 (38)	61 (26)	81 (54)	< 0.001*	< 0.001*	0.001*	0.007
**Healthy 55-year-old woman**	264 (97)	224 (97)	148 (99)	0.466	0.327	0.501	0.608
**80-year-old with ischemic cardiomyopathy who has dyspnoea with ordinary activity**	44 (16)	39 (17)	56 (37)	< 0.001*	< 0.001*	< 0.001*	0.904

All P values are two sided. * OBG compared to FP were significantly (P < 0.001).

Bivariate analysis showed that physicians beliefs of screening mammography in reducing breast cancer mortality for women aged 40-49 years were not associated with physicians gender, age, number of years in practice, family history of breast cancer or personal history of breast cancer among female physicians. However, the mean scores for response "very effective" were significantly higher for physicians of Asian race/ethnicity (*p *= < 0.01), those practicing in the South regions of the US (*p *= 0.05), and female physicians who had a mammogram (*p *= 0.03) (Additional File 1, Table S1).

The results of multivariate models (ordinal logistic regression analysis, and logistic regression) adjusted for primary care specialty, physician's race/ethnicity, and the US region are displayed in Table 3. OBG compared to FP had significantly higher odds for perceived effectiveness of mammography among women aged 40-49 and 70-89 years (OR 2.0, 95% CI: 1.3-3.1 and 4.4, 95% CI: 2.6-7.8, respectively). OBG were also more likely than FP to recommend mammography for women ages 40-49 (OR 3.0, 95% CI: 1.4-6.3), 50-69 years (OR 2.5, 95% CI: 1.1-5.9) and for older women age 70 or over (OR 4.5, 95% CI: 2.6-7.4) respectively. General internists did not differ significantly from family physicians in their ratings of the perceived effectiveness of mammography, and their recommendations for screening mammography.

**Table 3:** Primary care physicians' perceived effectiveness, and recommendations of screening mammography for average-risk women

	Model 1*	**Model 2 **^†^
**Perceived effectiveness (Ordinal logistic regression)**		

**1. "not effective", 2. "somewhat effective", 3. "very effective"**		
**Age 40-49 years**		
IM vs FP	1.06 (0.8-1.5)	0.9 (0.7-1.3)
OBG vs FP	1.98 (1.3-3.0)	2.0 (1.3-3.1)
**Age 50-69 years**		
IM vs FP	1.0 (0.6-1.7)	1.0 (0.6-1.7)
OBG vs FP	1.0 (0.6-1.9)	1.1 (0.6-2.7)
**Age 70-89 years**		
IM vs FP	1.4 (0.9-2.0)	1.3 (0.9-1.9)
OBG vs FP	4.3 (2.5-7.3)	4.5 (2.6-7.8)
**Recommendations for screening mammography (Logistic regression)**		
**Age 40-49 years: "always"/"often" vs "sometime"/"rarely"/"never"**		
IM vs FP	0.8 (0.5-1.3)	0.7 (0.5-1.2)
OBG vs FP	2.9 (1.4-6.2)	3.0 (1.4-6.3)
**Age 50-69 years: "always" vs "often"/"sometime"/"rarely"/"never"**		
IM vs FP	1.0 (0.6-1.7)	1.1 (0.6-1.9)
OBG vs FP	2.4 (1.04-5.7)	2.5 (1.1-5.9)
**Age 70-89: "always"/"often" vs "sometime"/"rarely"/"never"**		
IM vs FP	1.4 (0.9-1.2)	1.3 (0.9-1.9)
OBG vs FP	4. 3 (2.5-7.4)	4.5 (2.6-1.9)

*Model 1. Primary care physician specialty; comparing general internists and obstetricians and gynaecologists versus reference category family physicians.

^† ^Model 2. Adjusted for primary care physician specialty (reference category: FP), US region (reference category: Northeast), and race/ethnicity (reference category: white).

There were no differences in demographic and practice characteristics of physicians who responded to the first and second Med E-Mail Broadcast. Physicians who responded to third Med E-Mail Broadcast (third-wave respondents) compared to earlier respondents showed significant differences by demographic and practice characteristics. Early respondents (first and second wave) compared to third-wave respondents were more likely to be females compared to males (58% versus 44%) (*p *= < 0.001); 25-54 of age compared those age > = 55 years (84% versus 69%), OBG compared to FP (34% versus 23%), those reporting higher percent of new females patients seen per week (27% versus 26%) and physicians who were < = 9 years in practice (48% versus 38%) *P *= < 0.001. However, there were no differences in responses between early compared to late respondents in the physician's perceived belief in mammography effectiveness in reducing breast cancer mortality, responses to guideline influence, and recommendations for screening for women in different age categories.

## Discussion

In this cross sectional survey, the majority of PCPs believed in the effectiveness of mammogra*p*hy in reducing breast cancer mortality for women between 50 and 69 years of age, consistent with screening mammography consensus guidelines. However, we observed inconsistency between guideline influence and screening practices and recommendations. Despite higher endorsement of USPSTF guidelines by FP and IM than other guidelines, 84% of family physicians, 81% of internists reported recommending mammography to women aged 40-49 years. Overall, 15% of physicians would recommend mammography to a 50 year old woman with unresectable lung cancer and an 80 year old woman with advanced heart failure.

In comparisons across physician specialties, OBG indicated stronger belief in the effectiveness of mammography in reducing breast cancer mortality, and were more likely to recommend mammography, in younger (40-49 year old) and older (70 years or over) women (Figure 2). The propensity of OBG to endorse more aggressive screening may reflect ACS and ACOG guidelines, which recommend mammography every one to two years in women from 40 to 50 years of age and annually after age 50 with no specific age for cessation [5].

The association between stronger beliefs and recommendations for screening mammography by OBG compared to IM and FP in this study are in agreement with previous studies [17,20].

Discordance between endorsement of guidelines and actual practice has been observed in previous studies [2,21]. A possible explanation for this finding is that mammography for women aged 40-49 years is considered the standard of care in community practice and community physicians do not routinely act upon published guidelines in spite of their confidence in the guideline [2,21,22]. Clinicians may not fully appreciate that guidelines acknowledge the limited benefits of mammography in women aged 40-49 and > = 75 years and recommend that women at an average risk for breast cancer should make an informed decision with health care providers for breast cancer screening options based on patient preferences, health status and life expectancy.

This survey took place from June 2009 to December 2009. We observed differences in screening mammography recommendations between specialties before the release of the USPSTF update on breast cancer screening in November 2009 [4]. It is unclear how the revised USPSTF guidelines, which provoked considerable debate in the media, may have affected these differences. To the extent that USPSTF guidelines have more influence on the mammography recommendations of family physicians and general internists, compared with OBG, the observed differences in perceived effectiveness and recommendations for women aged 40-49 years in Table 3 may further increase after publication of the revised USPSTF guidelines. The revised guidelines did not address mammography use for older women or women with comorbid illnesses, so the analysis in Table 2 should not be affected.

Recommendations for breast cancer screening in the hypothetical case vignettes varied little across physician specialties based on the chronological age of patients; however, OBG were significantly more likely than IM and FP to recommend mammography in patients with limited life expectancy from coexisting comorbidities. Similar to our findings, previous studies have found that age is more important than comorbidities in influencing clinical decision-making [23,24].

These results may be explained in several ways. First, mixed strategies tailored to individuals' clinical characteristics and values are difficult to communicate and implement in public health practice. Second, mammography is a covered Medicare (fee-for-service) benefit [23] and is widely promoted, and accepted regardless of life expectancy and comorbidity. Third, physicians may anticipate that not recommending mammography to patients may decrease patient satisfaction, and increase potential liability, as reported previously [25].

Our study results are based on self-reported behaviours, and are thus likely to overestimate attitudes and clinical behaviours viewed as desirable in actual practice. Compared to third wave respondents early respondents were predominantly females compared to males, OBG compared to FP, 25-54 years compared to > = 55 years of age, reported seeing higher number of new patients, and were < = 9 years in practice. These results are different than reported from previous research on the web, postal and mixed surveys studies showing that male physicians are more likely to respond to surveys than female physicians [26]. This finding may be due to specific circumstances of this study, as breast cancer is the second leading cause of cancer in women in the US, controversies around regarding mammography guidelines, and OBG perceive themselves as primary care providers for women.

Our data are limited by the use of physicians' self reported preferences at a single point in time rather than measures of actual clinical decisions. One of the main limitations of this study is the low response rate (< 10%). This rate is not surprising given that response rates to surveys have declined dramatically over time due to proliferation of junk mail, rapid growth and ease of large-scale physician surveys, and resulting complaints as physicians feel "bombarded" with Internet based surveys despite increasing demands on their time [18]. We chose to sample PCPs from a technically literate sampling frame thus extrapolation of the results is limited as the respondents to our web-based survey may not represent the general population of PCPs [27]. Although initial response rate improved with a higher incentive, ($20 versus $5); however it is unlikely that sending additional email reminders or higher incentive would change our study results, or external validity [27]. Comparisons between early respondents and late respondents suggested non-response was associated with gender, physician specialty, age, and number of years in practice. However, the absence of any differences between early and late respondents on questions related to perceived effectiveness and mammography recommendations suggests that nonresponse did not substantively bias our key findings. Although we tried to explore the potential for non-response bias, this method still may not capture the true extent of non-response bias in our survey results [18,19,28].

Although case vignettes provided some background, for the physician respondent it was a hypothetical situation that does not reflect actual primary care practice, in which physicians provide continuing care of patients who have a variety of coexisting clinical issues. The magnitude of the influence of these factors may be considerably underestimated or overestimated with the use of clinical case vignettes.

Strengths of this study include a nationally representative sample of primary care specialties.

This study examined complex factors, and the importance of the cognitive component in mammography decision making-in particular in the face of uncertain guidelines among younger and older patients with specific clinical profiles. One of the key findings of this study is that primary care physicians' perceptions of the effectiveness of screening mammography is very important in decision making than the scientific evidence behind a guideline, additionally the presence of comorbidity, play a minor role.

## Conclusions

This study demonstrated high awareness of breast cancer screening in a geographically diverse sample of primary care physicians. However, knowledge gaps about the risks and benefits of screening mammography in younger and older women, including older women with comorbidities, are evident. Policy interventions that focus on potentially modifiable physician factors and cultures of practice change within primary care specialties are needed to reduce potential overuse of screening mammography. Although we studied primary care physicians at a single point in time and this survey reflects only the stated preferences of respondents, not their actual practices. However, our findings suggest that physicians' beliefs and perceptions of effectiveness influence their practice and recommendations for screening mammography. Differing preferences for clinical practice guidelines across specialties suggest that tailoring education by physician specialty may be more effective in reducing inter-specialty practice variation for screening mammography. Combinations of age and comorbidities should be included in refining guidelines for older women to help foster professional consensus about when to encourage or discourage mammography.

## Competing interests

The authors declare that they have no competing interests.

## Authors' contributions

SY, PSR and RLK participated in the study concept and design, acquisition of data, analysis and interpretation of data, drafting and critical revision of the manuscript, obtained funding, provided administrative, technical, or material support, and provided supervision. DJT participated in the study concept and design, analysis and interpretation of data, drafting and critical revision of the manuscript, and provided statistical expertise. NHS participated in the study design, critical revision of the manuscript, and provided statistical expertise. JR participated in the study concept and design, analysis and interpretation of data, critical revision of the manuscript, and provided administrative, technical, or material support of data, drafting of the manuscript, and provided administrative, technical, and material support. All authors read and approved the final manuscript.

## Pre-publication history

The pre-publication history for this paper can be accessed here:

http://www.biomedcentral.com/1472-6963/12/32/prepub

## Supplementary Material

Additional File 1 **Association of physician characteristics and effectiveness of screening mammography in reducing breast cancer mortality in different age categories**. The table shows the association of primary care physician's characteristics (race, gender, age, years in practice, US region of practice, personal history of having had a screening mammogram, personal history of breast cancer and family history of breast cancer) and their perceived effectiveness of screening mammography in reducing breast cancer mortality in women age 40-49, 50-69 and 70-89 years. Click here for file
