# Supplementary material for: Screening mammography beliefs and recommendations: a web-based survey of primary care physicians
Source: BMC Health Serv Res. 2012 Feb 6;12:32. doi: 10.1186/1472-6963-12-32 (PMC3293074; doi:10.1186/1472-6963-12-32)
Supplement: Additional File 1 — Association of physician characteristics and effectiveness of screening mammography in reducing breast cancer mortality in different age categories. The table shows the association of primary care physician's characteristics (race, gender, age, years in practice, US region of practice, personal history of having had a screening mammogram, personal history of breast cancer and family history of breast cancer) and their perceived effectiveness of screening mammography in reducing breast cancer mortality in women age 40-49, 50-69 and 70-89 years. [file 1472-6963-12-32-S1.DOC]

| **Table S1. Association of physician characteristics and effectiveness of screening mammography in reducing breast cancer mortality in different age categories** | | | |
| --- | --- | --- | --- |
| *** Mean value of 1: “not effective”, 2: “somewhat effective”, 3: “very effective”** | | | |
| **Age categories** | **40-49 years** | **50-69 years** | **70-89 years** |
| **Race** |  |  |  |
| White | 2.34 (n=490) | 2.85 (n=494) | 2.29 (n=460) |
| AA | 2.48 (n=23) | 2.83 (n=23) | 2.43 (n=21) |
| Hispanic | 2.53 (n=19) | 2.95 (n=19) | 2.33 (n=18) |
| Asian | 2.59 (n=99) | 2.96 (n=100) | 2.43 (n=90 ) |
| Other | 2.75 (n=8) | 2.78 (n=9) | 2.5 (n=8) |
| *ANOVA p-value* | *< 0.01* | *0.04* | *0.36* |
| **Gender** |  |  |  |
| Male | 2.36 (n=362) | 2.86 (n=363) | 2.29 (n=335) |
| Female | 2.44 (n=278) | 2.88 (n=283) | 2.36 (n=263) |
| *ANOVA p-value* | *0.1* | *0.53* | *0.21* |
| **Age** |  |  |  |
| 25-34 y/o | 2.38 (n=45) | 2.91 (n=46) | 2.22 (n=41) |
| 35-44 y/o | 2.40 (n=193) | 2.88 (n=196) | 2.31 (n=177) |
| 45-54 y/o | 2.46 (n=201) | 2.86 (n=204) | 2.38 (n=192) |
| 55-64 y/o | 2.32 (n=163) | 2.85 (n=163) | 2.25 (n=151) |
| 65 + y/o | 2.35 (n=37) | 2.92 (n=36) | 2.47 (n=36) |
| *ANOVA p-value* | *0.31* | *0.73* | *0.21* |
| **Years in Practice** |  |  |  |
| 1~9 yrs | 2.42 (n=248) | 2.87 (n=254) | 2.28 (n=234) |
| 10~20 yrs | 2.39 (n=229) | 2.88 (n=228) | 2.33 (n=209) |
| >=21 yrs | 2.36 (n=163) | 2.85 (n=164) | 2.36 (n=155) |
| *ANOVA p-value* | *0.62* | *0.74* | *0.51* |
| **US Region** |  |  |  |
| North East | 2.42 (n=165) | 2.83 (n=165) | 2.35 (n=161) |
| Mid West | 2.41 (n=137) | 2.91 (n=140) | 2.37 (n=127) |
| South | 2.47 (n=156) | 2.87 (n=157) | 2.38 (n=143) |
| West | 2.29 (n=182) | 2.88 (n=184) | 2.20 (n=167) |
| *ANOVA p-value* | *0.05* | *0.28* | *0.06* |
| **Had a mammogram (female only)** | |  |  |
| Yes | 2.50 (n=201) | 2.90 (n=202) | 2.39 (n=191) |
| No | 2.32 (n=75) | 2.84 (n=79) | 2.29 (n=70) |
| *ANOVA p-value* | *0.03* | *0.19* | *0.27* |
| **Personal history of breast CA (female only)** | | |  |
| Yes | 2.80 (n=10) | 3.0 (n=10) | 2.67 (n=9) |
| No | 2.43 (n=266) | 2.88 (n=271) | 2.35 (n=252) |
| *ANOVA p-value* | *0.07* | *0.27* | *0.18* |
| **Family history of breast CA** | |  |  |
| Yes | 2.37 (n=160) | 2.88 (n=162) | 2.36 (n=149) |
| No | 2.42 (n=305) | 2.86 (n=308) | 2.31 (n=283) |
| *ANOVA p-value* | *0.42* | *0.64* | *0.53* |

*P= < 0.05 (significant)*
